# Bedside monitoring of lung volume available for gas exchange

**DOI:** 10.1186/s40635-020-00364-6

**Published:** 2021-01-11

**Authors:** Minh C. Tran, Douglas C. Crockett, John N. Cronin, João Batista Borges, Göran Hedenstierna, Anders Larsson, Andrew D. Farmery, Federico Formenti

**Affiliations:** 1 Nuffield Division of Anaesthetics, University of Oxford, Oxford, UK; 2 Department of Engineering Science, University of Oxford, Oxford, UK; 3 Centre for Human and Applied Physiological Sciences, King’s College London, London, UK; 4 Department of Anaesthetics, Guy’s and St. Thomas’ NHS Foundation Trust, London, UK; 5 Hedenstierna Laboratory, Department of Medical Sciences, Uppsala University, Uppsala, Sweden; 6 Hedenstierna Laboratory, Department of Surgical Sciences, Uppsala University, Uppsala, Sweden; 7 Department of Biomechanics, University of Nebraska, Omaha, NE USA

**Keywords:** Computed tomography, Lung volume, Arterial oxygen partial pressure

## Abstract

**Background:**

Bedside measurement of lung volume may provide guidance in the personalised setting of respiratory support, especially in patients with the acute respiratory distress syndrome at risk of ventilator-induced lung injury. We propose here a novel operator-independent technique, enabled by a fibre optic oxygen sensor, to quantify the lung volume available for gas exchange. We hypothesised that the continuous measurement of arterial partial pressure of oxygen (PaO_2_) decline during a breath-holding manoeuvre could be used to estimate lung volume in a single-compartment physiological model of the respiratory system.

**Methods:**

Thirteen pigs with a saline lavage lung injury model and six control pigs were studied under general anaesthesia during mechanical ventilation. Lung volumes were measured by simultaneous PaO_2_ rate of decline (*V*_PaO2_) and whole-lung computed tomography scan (*V*_CT_) during apnoea at different positive end-expiratory and end-inspiratory pressures.

**Results:**

A total of 146 volume measurements was completed (range 134 to 1869 mL). A linear correlation between *V*_CT_ and *V*_PaO2_ was found both in control (slope = 0.9, *R*^2^ = 0.88) and in saline-lavaged pigs (slope = 0.64, *R*^2^ = 0.70). The bias from Bland–Altman analysis for the agreement between the *V*_CT_ and *V*_PaO2_ was − 84 mL (limits of agreement ± 301 mL) in control and + 2 mL (LoA ± 406 mL) in saline-lavaged pigs. The concordance for changes in lung volume, quantified with polar plot analysis, was − 4º (LoA ± 19°) in control and − 9° (LoA ± 33°) in saline-lavaged pigs.

**Conclusion:**

Bedside measurement of PaO_2_ rate of decline during apnoea is a potential approach for estimation of lung volume changes associated with different levels of airway pressure.

## Background

The acute respiratory distress syndrome (ARDS) is a common condition affecting about 100,000 patients yearly in the European Union and has a high mortality rate of ~ 40%, with ARDS patients occupying ~ 10% of intensive care unit beds in the UK [1]. ARDS patients require mechanical ventilation to maintain blood gases within the desired range. Despite low tidal volumes and limited plateau pressures being associated with better outcomes, this lifesaving mechanical ventilation can paradoxically contribute to the high mortality by further damaging the lung [2]. Clinical trials aimed at reducing lung injury via the application of high positive end-expiratory pressure (PEEP) levels have demonstrated conflicting results [3], suggesting that similar ventilator settings do not produce the same response at the alveolar level between different patients, especially in the presence of the “baby lung”, a smaller lung volume available for ventilation [4]. This could also be the case in the presence of lung heterogeneity like in COVID-19, where very similar arterial partial pressure of oxygen (PaO_2_) can be observed for very different computed tomography (CT) values [5–7]. There is a clear need for developments to enable individual titration of mechanical ventilation strategy, for example bedside real-time measurement of lung volume actually available for gas exchange, especially following changes between PEEP levels [8], which may contribute to the identification of patients with recruitable lungs.

Techniques currently available for the bedside measurement of lung volume are not routinely used because of different limitations including costs, physical dimensions, accuracy, time and conditions required for the measurement itself [9–11], although promising, operator-independent approaches are emerging [12–16].

In the presence of a single-compartment model of the lung, and for a known oxygen uptake, the rate of PaO_2_ decline during apnoea is inversely proportional to lung volume [17–19]. While the injured lung is highly heterogeneous in the dynamic state, this heterogeneity may be reduced after a few seconds of apnoea [20]. Continuous monitoring of PaO_2_ during breath-holding manoeuvres at the bedside may allow measurement of lung volume, and together with lung mechanics parameters, enable the calculation of lung strain ratio [21].

The aim of this study was to validate a novel method to determine lung volume available for gas exchange in real-time at the bedside by PaO_2_ rate of decline and oxygen uptake during apnoea. These measurements were performed at different positive end-expiratory and end-inspiratory pressures in mechanically ventilated pigs with saline lavage lung injury and in control pigs. Simultaneous whole-lung CT imaging data were used for comparison, where the lung volumes determined by CT would include regions with air trapped behind closed airways and aerated regions that are poorly perfused with blood, each of which would not contribute significantly to gas exchange, and could overlook regions with very limited aeration.

## Methods

### Ethical approval

The study was performed in a total of 19 domestic pigs (mean weight (SD) = 30 (2) kg) as part of different experiments at the Hedenstierna laboratory, Uppsala University, Sweden. The studies were approved by the regional animal welfare ethics committee (Ref: C98/16) and adhered to the Animal Research: Reporting of in vivo Experiments guidelines [22].

### Animal preparation and monitoring

The animals’ baseline characteristics are summarised in Table 1. Animals received intramuscular sedation and were anaesthetised with total intravenous anaesthesia as described elsewhere [23]. Mechanical ventilation was delivered at 20–25 breaths per minute with a tidal volume (*V*_T_) of 10 mL/kg and an inspiratory:expiratory ratio (I:E) of 1:2 (Servo-I, Maquet Critical Care, Solna, Sweden). Lack of spontaneous movements, absence of reaction to painful stimulation between the front hooves and absence of cardiovascular signs of sympathetic stimulation (increases in heart rate or arterial blood pressure) confirmed the depth of anaesthesia. Once anaesthesia was ascertained, rocuronium was administered for muscle relaxation. Continuous infusion of Ringerfundin™ solution (Braun Melsungen Ag, Melsungen, Germany) was used for fluid replacement at 10 mL/kg^/^h during the preparation and at 7 mL/kg^/^h thereafter. Cardiac output was monitored intermittently by pulmonary artery catheter thermodilution, together with arterio-mixed venous blood samples used to calculate oxygen uptake $\left({\dot{V}}_{{\mathrm{O}}_{2}}\right)$ via the Fick’s principle. Physiological parameters were continuously recorded with standard patient monitors (respiratory monitor: Datex Ohmeda Capnomac Ultima; multi-parameter patient monitor: Datex AS3) and electrical impedance tomography (EIT) to monitor pulmonary regional ventilation changes (Enlight, TIMPEL SA, São Paulo, Brazil). Analogue signals were continuously recorded on a computer via PowerLab (AD Instruments, New Zealand).

**Table 1:** Baseline characteristics for animals

Parameters	Control (*n* = 6)	Saline lavage lung injury model (*n* = 13)
Weight (kg)	31 (2)	30 (2)
Heart rate	97 (24)	91 (16)
SBP (mmHg)	111 (7)	100 (10)
DBP (mmHg)	71 (8)	59 (12)
Cardiac output (L/min)	3.9 (1.2)	3.4(0.6)
PASP (mmHg)	32 (5)	35 (5)
PADP (mmHg)	13 (6)	18 (6)
Hb (g/dL)	84 (9)	84 (7)
F_I_O_2_	0.4 (0.1)	0.8 (0.1)
SaO_2_ (%)	99 (1)	95 (7)
pH	7.42 (0.03)	7.28 (0.09)
PaO_2_ (kPa)	19.9 (3.2)	16.4 (7.5)
PaCO_2_ (kPa)	6.1 (0.8)	8.5 (2.1)
PFR (mmHg)	425 (100)	160 (84)

Control animals and saline lavage lung injury model, mean (SD) are shown

SBP, systolic blood pressure; DBP, diastolic blood pressure; PASP, pulmonary artery systolic pressure; PADP, pulmonary artery diastolic blood pressure; Hb, haemoglobin; F_I_O_2_, fraction of inspired O_2_; SaO_2_, arterial oxygen saturation; PaO_2_, arterial O_2_ partial pressure; PaCO_2_, arterial CO_2_ partial pressure; PFR, PaO_2_:F_I_O_2_ ratio

### Saline lavage lung injury model

For this initial proof-of-concept study, a collapse-prone lung injury was induced with a technique modified from Lachmann and colleagues [24]. Mechanical ventilation with a fraction of inspired O_2_ (F_I_O_2_) of 1.0 preceded the disconnection of the ventilator and surfactant depletion via lung lavage with 30 mL/kg of 0.9% saline solution (at 37 °C) instilled via the tracheal tube. After 30 s, saline was drained out of the lungs and ventilation recommenced. This process was repeated until the PaO_2_:F_I_O_2_ ratio (PFR) < 300 mmHg (40 kPa) was achieved at a PEEP of 5 cmH_2_O and F_I_O_2_ of 0.7.

### Study protocol

A series of 20 s breath-holding manoeuvres was performed at different PEEP levels [range 0 to 20 cmH_2_O] and at end-inspiratory pressures associated with a *V*_T_ of 10 mL/kg. Whole lung CT scans and continuous PaO_2_ data were recorded simultaneously during the breath-holding manoeuvres.

### CT image acquisition and analysis

CT images were acquired with a Somatom Definition Flash (Siemens, Erlangen, Germany), used as a gold standard to measure lung volumes. Tube voltage was 80 kV, with 364 mA current and 64 × 60 mm collimation. Reconstituted voxel dimensions were 0.5 × 0.5 × 5 mm.

CT image analysis was performed using 3D Slicer v4.10.2 [25] (https://www.slicer.org), as presented elsewhere [26]. Aerated lung volume was calculated according to mean Hounsfield unit (HU) density within the segmented lung volume.

The HU boundaries were water with a density of 1 g cm^−3^ (0 HU) and air with a density of 0 g cm^−3^ (− 1000 HU). Gas volume was computed as in Chiumello et al. [27]:

$${V}_{\mathrm{CT}}= \frac{-\mathrm{CT} \,\mathrm{density} (\mathrm{HU})}{1000} \times \mathrm{segmented\, lung \,volume}.$$

### Continuous measurement of PaO_2_ and mathematical modelling

PaO_2_ was recorded with a fibre optic sensor based on luminescence quenching by oxygen of a fluorophore embedded in a polymer material, polymethyl-methacrylate [23, 28–31], inserted in a carotid artery via a standard arterial catheter. The sensor has a response time ~ 100 ms and data were recorded continuously at 10 Hz. Technical properties of this sensor are presented elsewhere [30, 32].

**Fig. 1:**
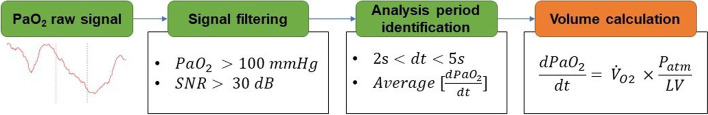
Algorithm to calculate lung volume available for gas exchange. The text in the coloured boxes summarises the main steps in the algorithm leading to the equation used for the calculation of lung volume available for gas exchange. The text in the white boxes presents the physiological and signal conditions imposed a priori for the automatic processing and analysis of the raw PaO_2_ signal, necessary for a bias-free selection of the representative period that was used for the automatic calculation of lung volume available for gas exchange. Additional methodological details are presented in the methods. PaO_2_ arterial partial pressure of oxygen; SNR: signal-to-noise ratio; *V̇*_O2_: oxygen uptake; *P*_atm_: atmospheric pressure; LV: lung volume

**Fig. 2:**
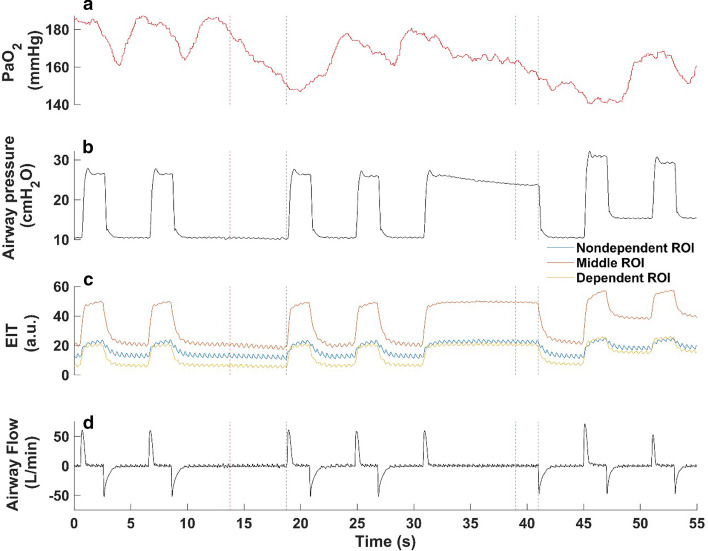
Representative responses to breath-holding manoeuvres performed during expiration and inspiration. **a** The arterial partial pressure of oxygen (PaO_2_), **b** the airway pressure, **c** the electrical impedance tomography (EIT) for three gravitational regions of interest (ROI), and **d** the airway flow. The vertical dashed red and black lines present the start and end points of the period used for analysis; minimum and maximum durations of this period were 2 and 5 s, depending on linearity of the PaO_2_ signal (see “Methods” for details)

As illustrated in Fig. 1, the lung was assumed to perform as a single compartment with a constant oxygen uptake. Here the rate of PaO_2_ decline is inversely proportional to lung volume, and directly proportional to the rate of oxygen uptake by the pulmonary circulation [17, 18]. Based on the equation presented in Fig. 1, predicted lung volume available for gas exchange can be calculated on the basis of measured oxygen uptake and rate of PaO_2_ decline during each breath-hold manoeuvre. The single compartment assumption was supported by stable airway pressure and flow, and EIT signal during the relevant period of apnoea, as illustrated in Fig. 2. In this single compartment assumption, the rate of alveolar oxygen change equals the difference between the rates of input via ventilation during inspiration and output via continuous uptake by the pulmonary circulation and elimination during expiration [19]. Pigs’ metabolism (e.g. heart rate, respiration and oxygen uptake) was stable during the experimental protocol.

The novel PaO_2_ signal processing technique proposed here is based on the calculation of PaO_2_ gradient between two random time points in the PaO_2_ signal during the overall breath-hold period, defined as the period with no airway flow. This iterative analysis was repeated a hundred times for each of the breath-hold manoeuvres, leading to the algorithm proposed here (Fig. 1), which identifies the period of linear decline on which the calculations to estimate lung volume available for gas exchange were based.

The linear PaO_2_ signal recorded in the last 2–5 s of each breath-holding manoeuvre was used to calculate the lung volume; an example is illustrated in Fig. 2. The airway flow, pressure and EIT signals were stable in the periods considered for analysis. The maximum analysis period was limited to 5 s to exclude transient changes in pulmonary ventilation, perfusion and their distributions, which might have occurred at the beginning of the breath-holding manoeuvres. These transient changes could have temporarily affected the linearity of the PaO_2_ rate of decline and would be overlooked by a single-compartment model of the respiratory system, hence would affect the accuracy of lung volume calculation. If the PaO_2_ decline was linear for 5 s, then a 5-s period was used to calculate the lung volume. Alternatively, a period shorter than 5 s was used for analysis, where a minimum period of at least 2 s was required because shorter periods were associated with relatively low signal-to-noise ratios and reduced accuracy and confidence. Breath-holding manoeuvres where PaO_2_ signal-to-noise ratio was < 30 dB were excluded from the analysis, as were those manoeuvres when PaO_2_ signal was smaller than 100 mmHg because of haemoglobin desaturation, which itself reduces the rate of PaO_2_ decline. For these two conditions combined, 20% of the data were excluded. Overall, this standardised procedure first filtered the raw signal to exclude cases where low signal-to-noise ratio and haemoglobin saturation reduction could have confounded the analysis, and then automatically calculated lung volume available for gas exchange, using an operator- and patient-independent approach to avoid subjectivity and bias.

### Statistical analysis

Linear regression, Bland–Altman and polar plots were used to assess the relationship and agreement between absolute lung volumes and their changes measured with CT and with PaO_2_ rate of decline [33, 34]. The linear relationship between measurements was analysed using linear mixed effects modelling with variation caused by different animals considered as a random effect. Conditional *R*^2^ values based on the entire model are reported [35]. Change in lung volumes (Δ*V*) were calculated from their values measured at different airway pressure levels, and compared using four-quadrant and polar plots [36], where concordance was assumed adequate within ± 30º of the horizontal. Statistical analyses were performed in Matlab v2018b (Mathworks, MA, USA).

## Results

A total number of 146 paired lung volume measurements were analysed in 6 control pigs and 13 saline lavage lung injury pig models at five different PEEP levels.

**Fig. 3:**
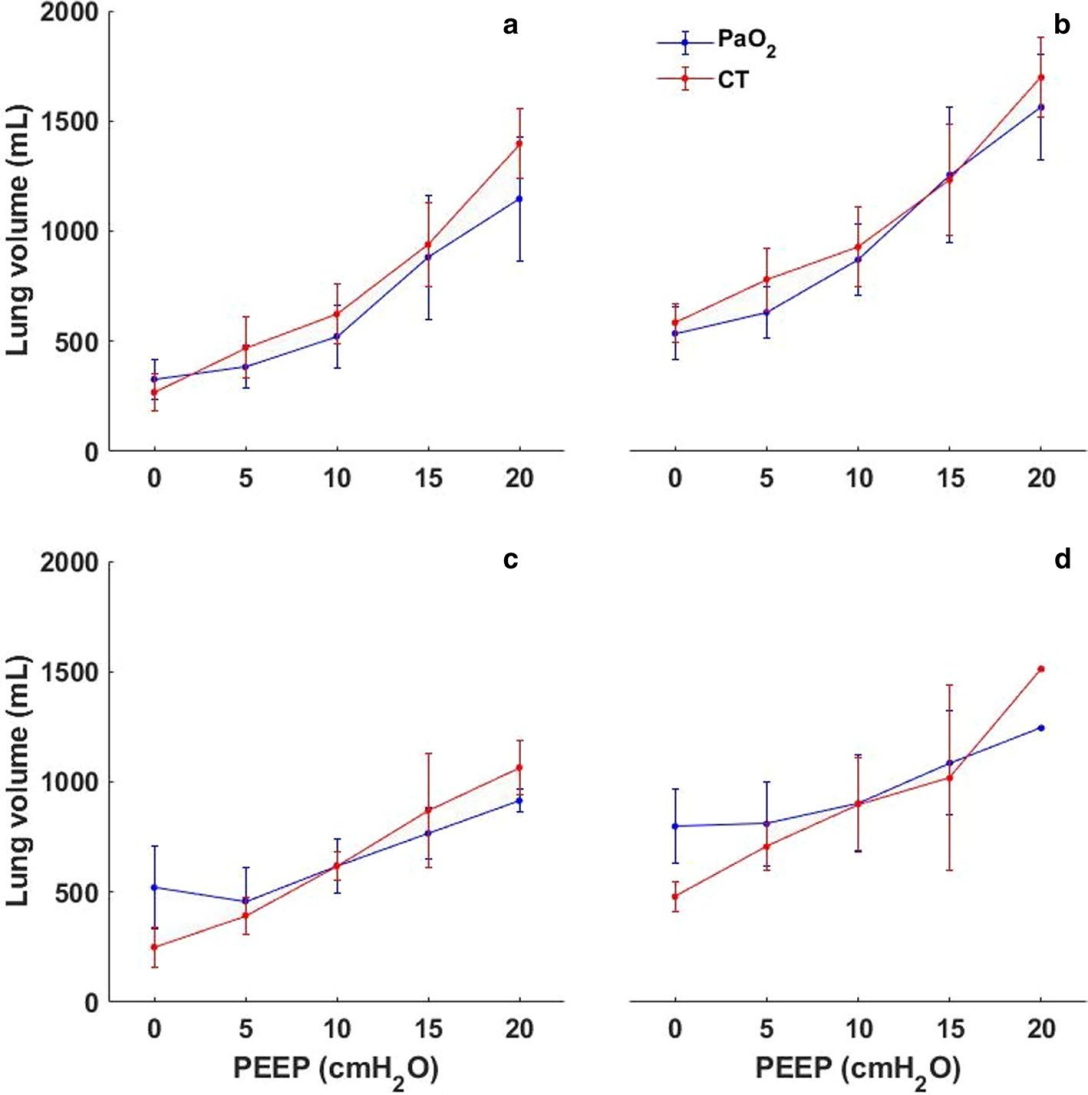
Lung volume measurement (mean ± SD) from PaO_2_ analysis and CT imaging at different PEEP levels during incremental PEEP titration. *V*_PaO2_ and *V*_CT_ measurements in the control animals during **a** apnoea at end-expiration and **b** end-inspiration. *V*_PaO2_ and *V*_CT_ measurements in the saline lavage lung injury model during **c** apnoea at end-expiration and **d** end-inspiration. Additional file 1: Table S2 provides full details of volumes measured and probabilities of difference

Figure 3 shows lung volumes and their PEEP-associated changes measured by PaO_2_ and CT. Overall, lung volumes available for gas exchange measured by PaO_2_ rate of decline (*V*_PaO2_) and those measured by whole-lung CT (*V*_CT_) increased with PEEP in the saline lavage model and in control animals. *V*_PaO2_ were greater than *V*_CT_ at PEEP 0 cmH_2_O in the saline lavage lung injury model during end-expiratory (volume difference = 270(96) mL) and the associated end-inspiratory (volume difference = 319(234) mL) breath-holding manoeuvres. Additional file 1: Table S1 details these *V*_PaO2_ and *V*_CT_ and their differences. Additional file 1: Table S2 shows details of multiple replicate measurements and that the mean coefficient of variation for V_PaO2_ repeated measurements within animal was less than 10% in both the control animals and in the saline lavage lung injury model.

### Absolute lung volumes

**Fig. 4:**
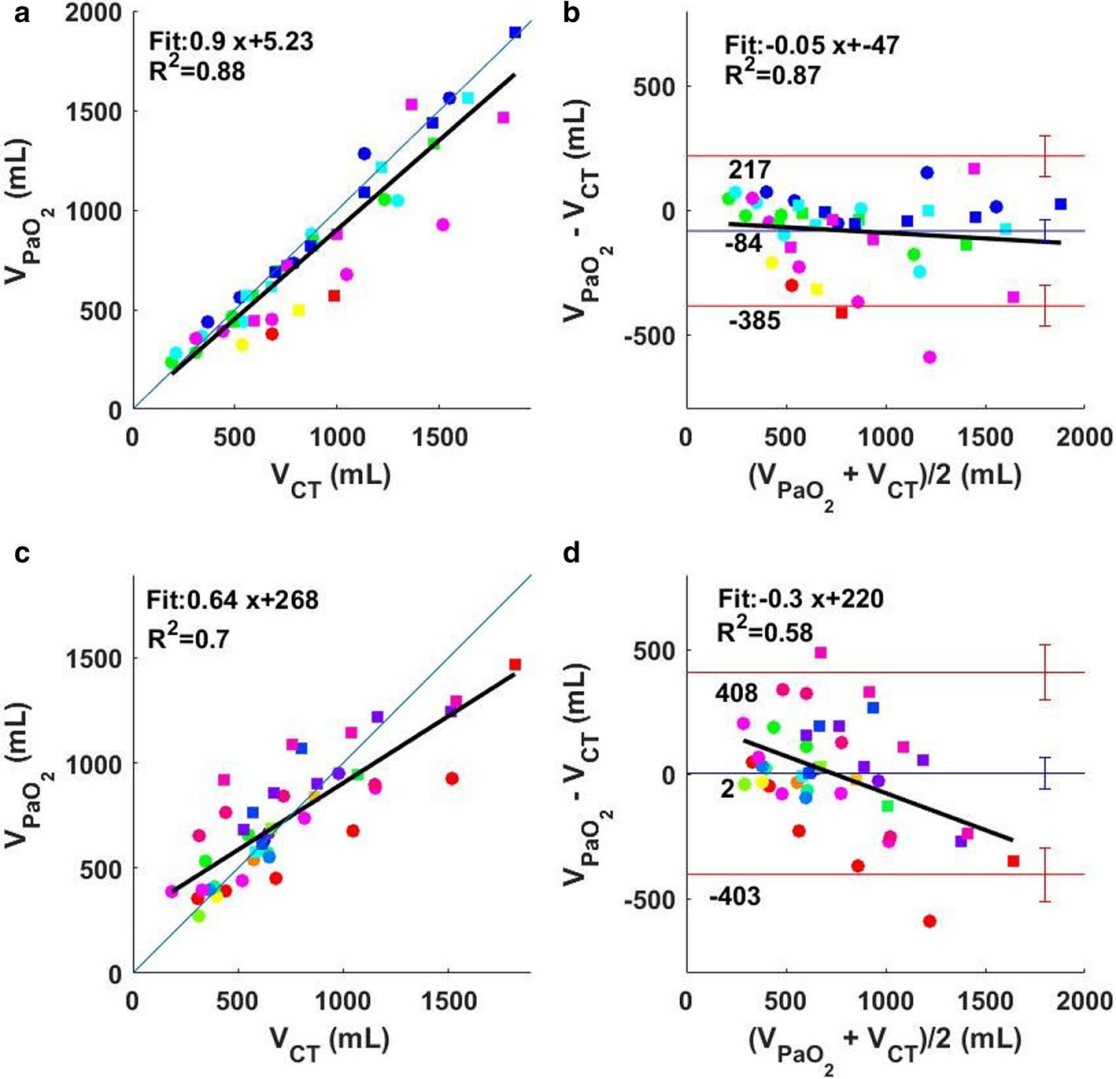
Linear regression and Bland–Altman analyses for absolute volume measurements of *V*_PaO2_ and *V*_CT_ in the control animals (**a**, **b**), and in the saline lavage lung injury model (**c**, **d**). Individual points represent a paired set of measurements at different PEEP levels (individual values in Additional file 1: Table S1). Each colour shows results from one animal. Circles and squares represent volumes measured, respectively, during end-expiratory and end-inspiratory apnoea. **a**, **c** Black solid lines are the regression lines, and the blue lines are the identity lines. **b**, **d** Black solid lines are the Bland–Altman plots’ regression lines, blue lines are the mean bias and red lines are the upper and lower limits of agreement (± 1.96 SD) with 95% confidence interval

Figure 4 shows the positive correlations between V_PaO2_ and V_CT_. Correlation values were 0.90 and 0.64, and R^2^ were 0.88 and 0.70, respectively, in the control pigs (Fig. 4a) and in the saline lavage lung injury model (Fig. 4c). The mean bias (± 95% limits of agreement) for V_PaO2_ and V_CT_ was − 84 mL (± 301 mL) in control pigs (Fig. 4b), and 2 mL (± 405 mL) in the saline lavage lung injury model (Fig. 4d), where the mean bias tended to decrease at larger lung volumes.

### Lung volume changes

**Fig. 5:**
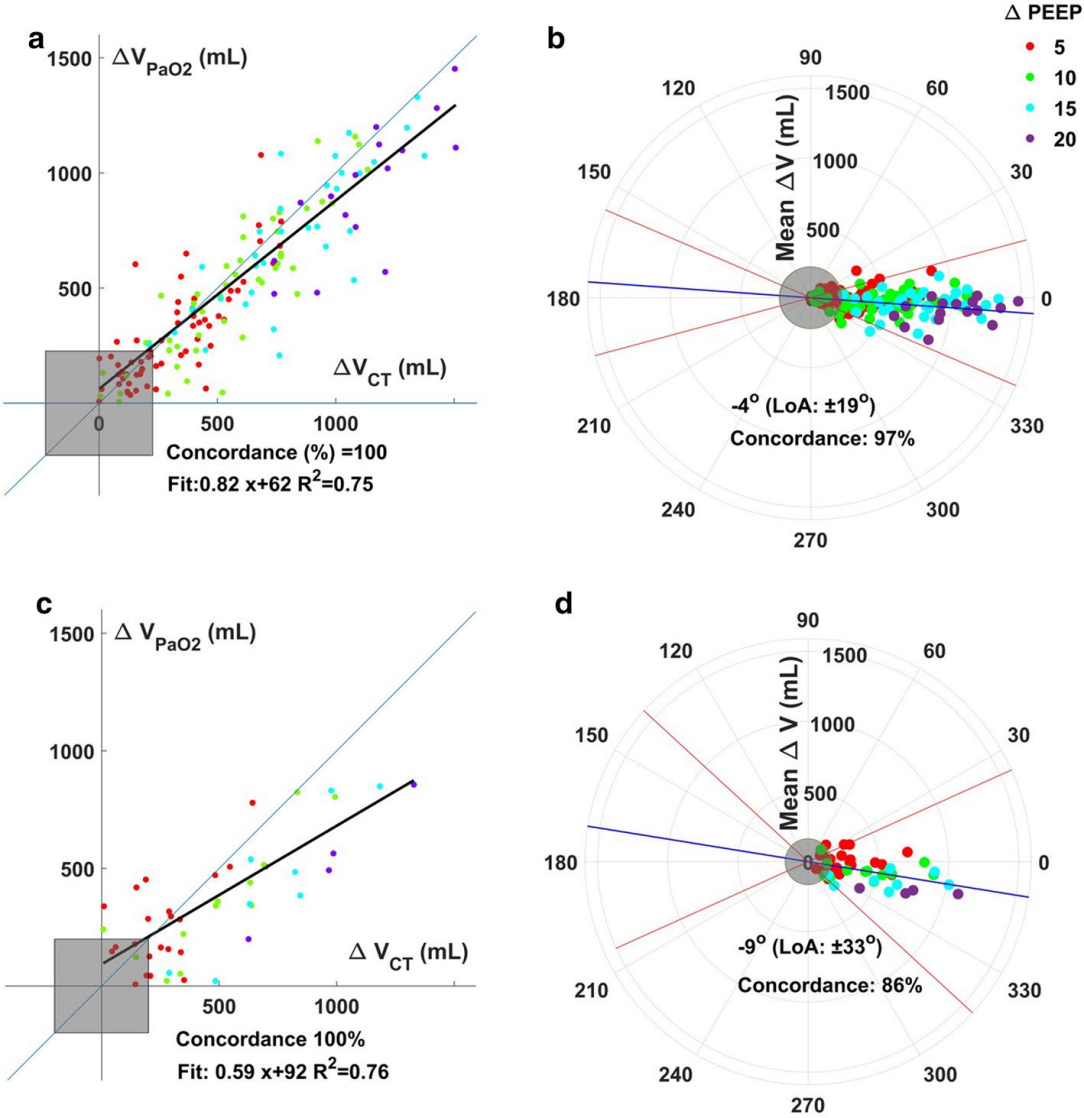
Volume changes (Δ*V*_PaO2_ and Δ*V*_CT_) in the control animals (**a**, **b**), and in the saline lavage lung injury model (**c**, **d**). Individual points represent a paired set of measurements and different colours represent changes associated with different ΔPEEP in each animal. **a**, **c** Black solid lines are the regression lines, and blue lines are the identity lines. **b**, **d** The stronger the agreement between two measurements, the closer the data are to the horizontal line (0°). **b**, **d** Blue lines are the mean angular bias, and red lines are the upper and lower limits of agreement; good concordance is assumed to be within ± 30° of the horizontal zero line. *LoA* limits of agreement

Figure 5 shows 100% concordance and positive correlations between Δ*V*_PaO2_ and Δ*V*_CT_. Correlation values were 0.82 and 0.59, and R^2^ were 0.75 and 0.76, respectively, in the control pigs (Fig. 5a) and in the saline lavage lung injury model (Fig. 5c). The mean angular bias (± 95% radial limits of agreement) for polar agreement between *V*_PaO2_ and *V*_CT_ was − 4° (± 19°), with a concordance of 97% in the control pigs (5b), and − 9° (± 33°), with a concordance of 86% in the saline lavage lung injury model (5d).

## Discussion

This pre-clinical study presents a novel bedside method to monitor lung volume available for gas exchange, and it demonstrates the positive correlation, agreement and small bias between *V*_PaO2_ and *V*_CT_ in a mechanically ventilated pig saline lavage lung injury model and in control pigs during breath-holding manoeuvres. While these results were associated with relatively small limits of agreement at low lung volumes, the limits of agreement indicated reduced accuracy at larger lung volumes, outside the < 5% recommendation [37]. The relatively smaller radial limits of agreement indicated a greater accuracy in the measurements of Δ*V*_PaO2_ and Δ*V*_CT_. *V*_PaO2_ results variability within animal was < 10%, within the reported acceptable limits of coefficient of variation [38].

The single-compartment model assumes that the air volume measured contributes to gas exchange in the lung (i.e. ventilated and perfused alveolar volume), whereas the air volume measured with CT imaging includes dead spaces. It is possible that this difference between techniques has contributed to the observed CT overestimation of absolute lung volume, especially at larger volumes in the saline lavage lung injury model, where greater volumes of air trapped behind closed airways may be present [39], as well as gas within conducting airways. At low lung volumes, especially at PEEP 0 cmH_2_O after saline lavage, it is possible that the HU range for non-aerated lung included a portion of lung that had at least a degree of ventilation and perfusion, hence contributed to gas exchange. Here, CT may not clearly distinguish between atelectatic regions not contributing to gas exchange, and regions with a very low ventilation and perfusion ratio (*V*/*Q*). For example, *V*_CT_ at PEEP 5 cmH_2_O could include volumes from the ideal alveolus and regions with a high *V*/*Q* (i.e. alveolar dead space), while *V*_PaO2_ would include volumes from the ideal alveolus and regions with a low *V*/*Q*. When PEEP is reduced to 0 cmH_2_O, *V*_CT_ would be reduced both by the loss of high V/Q regions, as well as by the greater proportion of atelectasis and low *V*/*Q* regions. In contrast, *V*_PaO2_ would still capture both ideal alveolus and also regions with low *V*/*Q*, possibly explaining the greater *V*_PaO2_ compared with *V*_CT_. This difference between techniques was smaller when considering changes in lung volume that may be important when determining, for example, the effect of a recruitment manoeuvre on lung volume.

The main limitations of the *V*_PaO2_ technique include the requirement for the brief ~20-s interruption of mechanical ventilation, the assumption that the lung can be mathematically modelled as a single compartment, for PaO_2_ to be greater than 100 mmHg, for an accurate measurement of oxygen uptake, and for a signal-to-noise ratio > 30 dB.

The *V*_PaO2_ technique must be performed during a breath-holding manoeuvre because rapid pulmonary ventilation and perfusion changes occur dynamically during tidal breathing, when the lung cannot be modelled as a single compartment. In this sense, a breath-holding manoeuvre shorter than ~ 20 s may be affected by transient and regional changes in pulmonary perfusion and ventilation, associated with pendelluft [40, 41]. Similarly long (or even longer) end-inspiratory breath-holding manoeuvres may be performed for recruitment in ARDS patients [42], but end-expiratory breath-holding manoeuvres, even at PEEP 5 cmH_2_O as in our study here, may increase the risk of greater lung collapse.

While PaO_2_ is normally greater than 100 mmHg in healthy, anaesthetised patients, it is typically lower in ARDS patients, where titration of mechanical ventilation is particularly important [2]. In order to reduce the chances of lung volume underestimation caused by haemoglobin desaturation, mean PaO_2_ would need to be raised above 100 mmHg for a few minutes, also as a safety precaution, for the duration of the breath-holding manoeuvre required for the measurement of lung volume via PaO_2_ rate of decline. This increase in PaO_2_ via greater F_I_O_2_ may not always be readily achievable.

Oxygen uptake was calculated from intermittent cardiac output measurements with thermodilution and from simultaneous arterio-venous blood samples [43]. In our study, a single oxygen uptake level was used to calculate *V*_PaO2_ across different breath-holding manoeuvres in order to limit the volume of saline injections and blood samples over the ~ 2-h experimental period. This approach could have contributed to the observed *V*_PaO2_ variability due to potentially overlooked changes in oxygen uptake, especially at higher lung volumes (end-inspiratory breath holds) in the saline lavage lung injury model. Here, greater positive airway pressure could have redistributed pulmonary perfusion [44] and reduced cardiac output during the breath-holding, hence reducing pulmonary oxygen uptake, possibly leading to an underestimation of lung volume. A greater accuracy would be expected with appropriate data collection timing, such as simultaneous measurement of oxygen uptake and *V*_PaO2_.

The fibre optic oxygen sensors used for our study are not yet produced in large quantities, hence their signal-to-noise ratio may be variable. Sensors’ positional changes within the arterial vessel could also reduce the signal-to-noise ratio, although these changes are unlikely to occur. Further developments in the manufacturing process of these prototype sensors are likely going to improve signal-to-noise ratio and generate more accurate *V*_PaO2_ measurements.

The main advantages of the *V*_PaO2_ technique include its potential use at the bedside, availability of lung volume measurement outcome within minutes, limited costs, and operator- and patient-independent results. The *V*_PaO2_ technique was more sensitive to relative than to absolute estimation of lung volume available for gas exchange. From a clinical perspective, this sensitivity may help distinguish recruitable from non-recruitable lung.

## Conclusion

In conclusion, lung volumes estimated from the PaO_2_ rate of decline during breath-holding manoeuvres correlated with volumes measured by CT, but with large limits of agreement due to several confounders. The validity of this novel method needs to be confirmed in other lung injury models before it is used clinically.

## Supplementary Information

**Additional file 1: Table S1. **Lung volume (upper part) and its changes (lower part) in both the control animals and the saline lavage lung injury model. **Table S2. **Mean (standard deviation) of the replicated volume measurements in 9 animals. An additional 50 measurements were performed, but data were not analysed due to either PaO_2_ < 100 mmHg, or signal-to-noise ratio < 30 dB. *n*: number of observations.

## Data Availability

Data are available upon reasonable request.
