# Supplementary material for: Bedside monitoring of lung volume available for gas exchange
Source: Intensive Care Med Exp. 2021 Jan 11;9:3. doi: 10.1186/s40635-020-00364-6 (PMC7835652; doi:10.1186/s40635-020-00364-6)
Supplement: Supplementary file 1 — Additional file 1: Table S1. Lung volume (upper part) and its changes (lower part) in both the control animals and the saline lavage lung injury model. Table S2. Mean (standard deviation) of the replicated volume measurements in 9 animals. An additional 50 measurements were performed, but data were not analysed due to either PaO2 < 100 mmHg, or signal-to-noise ratio < 30 dB. n: number of observations. [file 40635_2020_364_MOESM1_ESM.docx]

**Additional file 1: Table S1: Lung volume (upper part) and its changes (lower part) in both the control animals and the saline lavage lung injury model.**

| **PEEP**  **(cmH_2_O)** | **Control** (mL) | | | | | | | | **Saline lavage lung injury model** (mL) | | | | | | | |
| --- | --- | --- | --- | --- | --- | --- | --- | --- | --- | --- | --- | --- | --- | --- | --- | --- |
|  | **End Expiratory** | | | | **End Inspiratory** | | | | **End Expiratory** | | | | **End Inspiratory** | | | |
|  | **n** | **V_CT_** | **V_PaO2_** | **Mean difference** | **n** | **V_CT_** | **V_PaO2_** | **Mean difference** | **n** | **V_CT_** | **V_PaO2_** | **Mean difference** | **n** | **V_CT_** | **V_PaO2_** | **Mean difference** |
| **0** | 4 | 269(83) | 327(89) | 59(14) | 4 | 585(87) | 536(121) | -49(74) | 2 | 250(92) | 521(188) | 270(96) | 2 | 479(68) | 799(167) | 319(234) |
| **5** | 6 | 471(139) | 384(95) | -87(139) | 6 | 781(142) | 632(116) | -149(170) | 8 | 394(83) | 459(151) | 65(128) | 5 | 706(107) | 810(193) | 104(152) |
| **10** | 4 | 623(136) | 522(141) | -101(91) | 4 | 930(182) | 871(164) | -59(46) | 7 | 617(65) | 620(123) | 2(89) | 4 | 899(208) | 902(218) | 3(98) |
| **15** | 4 | 939(191) | 882(283) | -57(220) | 4 | 1233(254) | 1256(305) | 23(96) | 3 | 870(257) | 767(118) | -103(139) | 4 | 1018(422) | 1086(234) | 69(223) |
| **20** | 4 | 1398(159) | 1147(283) | -251(252) | 4 | 1698(181) | 1563(238) | -135(157) | 2 | 1065(123) | 915(49) | -150(172) | 1 | 1513 | 1244 | 269 |
|  |  |  |  |  |  |  |  |  |  |  |  |  |  |  |  |  |
| **Δ5** | 16 | 282(132) | 205(147) | -78(118) | 16 | 278(141) | 265(171) | -14(136) | 11 | 261(91) | 133(92) | -128(106) | 9 | 258(132) | 171(138) | -87(207) |
| **Δ10** | 12 | 555(191) | 433(228) | -122(150) | 12 | 541(205) | 534(207) | -7(110) | 5 | 513(156) | 231(158) | -281(172) | 5 | 573(161) | 272(73) | -301(185) |
| **Δ15** | 8 | 833(194) | 650(220) | -183(212) | 8 | 812(216) | 801(227) | -11(177) | 3 | 763(115) | 359(121) | -403(165) | 3 | 861(234) | 433(91) | -428(316) |
| **Δ20** | 4 | 1129(80) | 819(229) | -310(244) | 4 | 1027(126) | 1113(105) | -86(93) | 1 | 967 | 492 | -475 | 1 | 986 | 563 | -423 |
| Mean (SD) volumes are shown in mL. PEEP = Positive end-expiratory ratio, n = number of animals. V_PaO2_ = PaO_2_ measured absolute volume, V_CT_ = CT measured absolute volume. Δ = PEEP change, Mean difference = V_PaO2_ – V_CT_. | | | | | | | | | | | | | | | | |

**Additional file 1: Table S2:** Mean (standard deviation) of the replicated volume measurements in 9 animals. An additional 50 measurements were performed, but data were not analysed due to either PaO_2_ <100 mmHg, or signal-to-noise ratio <30dB. n: number of observations.

|  |  | **End expiration** (mL) | | | **End inspiration** (mL) | | |
| --- | --- | --- | --- | --- | --- | --- | --- |
| **Animal number** | **PEEP** (cmH_2_O) | **n** | **V_CT_** | **V_PaO2_** | **n** | **V_CT_** | **V_PaO2_** |
| **1** | 5 | 10 | 681(17) | 378(24) | 7 | 985(32) | 572(22) |
| **2** | 5 | 5 | 535(10) | 324(27) | 5 | 812(17) | 495(15) |
| **7** | 5 | 6 | 573(15) | 539(47) | 6 | 863(24) | 841(37) |
| **8** | 5 | 6 | 397(8) | 364(27) | 6 | 658(8) | 688(62) |
| **9** | 5 | 4 | 664(22) | 688(215) | 4 | 1064(94) | 832(319) |
| **10** | 5 | 6 | 313(5) | 272(46) | 5 | 583(5) | 575(62) |
| **11** | 5 | 2 | 345(1) | 536(4) |  | _ | _ |
|  | 10 | 2 | 541(8) | 677(19) |  | _ | _ |
| **12** | 5 | 2 | 390(2) | 413(3) |  | _ | _ |
|  | 10 | 2 | 633(8) | 545(29) |  | _ | _ |
| **14** | 5 | 2 | 359(5) | 390(6) |  | _ | _ |
|  | 10 | 2 | 669(21) | 546(6) |  | _ | _ |
